# A Culturally Adapted Diet and Physical Activity Text Message Intervention to Prevent Type 2 Diabetes Mellitus for Women of Pakistani Origin Living in Scotland: Formative Study

**DOI:** 10.2196/33810

**Published:** 2023-09-15

**Authors:** Marta Krasuska, Emma M Davidson, Erik Beune, Anne Karen Jenum, Jason MR Gill, Karien Stronks, Irene GM van Valkengoed, Esperanza Diaz, Aziz Sheikh

**Affiliations:** 1 Usher Institute University of Edinburgh Edinburgh United Kingdom; 2 Centre for Clinical Brain Sciences University of Edinburgh Edinburgh United Kingdom; 3 Department of Public and Occupational Health Amsterdam Public Health Research Institute University of Amsterdam Amsterdam Netherlands; 4 General Practice Research Unit (AFE), Department of General Practice, Institute of Health and Society Faculty of Medicine University of Oslo Oslo Norway; 5 Institute of Cardiovascular and Medical Sciences University of Glasgow Glasgow United Kingdom; 6 Department of Global Public Health and Primary Care University of Bergen Bergen Norway

**Keywords:** diabetes, diet, ethnic minority populations, Pakistani, physical activity, prevention, South Asian, text messages, women, women’s health, health intervention, digital health, mobile health, minority, exercise, text message, text messaging, SMS, development, formative, diabetes mellitus

## Abstract

**Background:**

Individuals of South Asian origin are at an increased risk of developing type 2 diabetes mellitus (T2DM) compared with other ethnic minority groups. Therefore, there is a need to develop interventions to address, and reduce, this heightened risk.

**Objective:**

We undertook formative work to develop a culturally adapted diet and physical activity text message intervention to prevent T2DM for women of Pakistani origin living in Scotland.

**Methods:**

We used a stepwise approach that was informed by the Six Steps in Quality Intervention Development framework, which consisted of gathering evidence through literature review and focus groups (step 1), developing a program theory for the intervention (step 2), and finally developing the content of the text messages and an accompanying delivery plan (step 3).

**Results:**

In step 1, we reviewed 12 articles and identified 3 key themes describing factors impacting on diet and physical activity in the context of T2DM prevention: knowledge on ways to prevent T2DM through diet and physical activity; cultural, social, and gender norms; and perceived level of control and sense of inevitability over developing T2DM. The key themes that emerged from the 3 focus groups with a total of 25 women were the need for interventions to provide “friendly encouragement,” “companionship,” and a “focus on the individual” and also for the text messages to “set achievable goals” and include “information on cooking healthy meals.” We combined the findings of the focus groups and literature review to create 13 guiding principles for culturally adapting the text messages. In step 2, we developed a program theory, which specified the main determinants of change that our text messages should aim to enhance: knowledge and skills, sense of control, goal setting and planning behavior, peer support, and norms and beliefs guiding behavior. In step 3, we used both the intervention program theory and guiding principles to develop a set of 73 text messages aimed at supporting a healthy diet and 65 text messages supporting increasing physical activity.

**Conclusions:**

We present a theory-based approach to develop a culturally adapted diet and physical activity text message intervention to prevent T2DM for women of Pakistani origin living in Scotland. This study outlines an approach that may also be applicable to the development of interventions for other ethnic minority populations in diverse settings. There is now a need to build on this formative work and undertake a feasibility trial of a text message–based diet and physical activity intervention to prevent T2DM for women of Pakistani origin living in Scotland.

## Introduction

### Background

The prevalence of type 2 diabetes mellitus (T2DM) has increased rapidly in recent decades such that it now represents a leading cause of morbidity and mortality [1,2], adversely affecting the health and well-being of those living with the condition [3] and causing a substantial burden on health systems worldwide [4,5]. Individuals from South Asian backgrounds, including those from Pakistan, India, Bangladesh, and Sri Lanka, are disproportionately affected by this growing epidemic. The increased risk of T2DM is especially high for South Asians living outside the Indian subcontinent who, on average, are 2 to 4 times more likely to have been diagnosed with T2DM [6,7] and tend to develop T2DM at a lower BMI [8,9] and 5 to 10 years earlier [10,11] compared with people from White European backgrounds living in the same countries. The specific factors leading to this high prevalence of diabetes remain unclear; however, a number of factors have been hypothesized to contribute to this markedly increased risk, including diet, levels of physical activity, social factors, broader environmental factors, and likely a complex interplay between all the aforementioned etiologies [12,13]. An in-depth exploration of this high susceptibility to T2DM, and coronary heart disease, included evidence from 22 international experts and concluded that this remains a complex matter that could not yet be fully explained [14]. However, despite the current state of this research and the emergence of newer ideas needing further exploration (eg, high-heat cooking or microcirculatory damage) [14], influencing the modifiable risk factors such as diet and physical activity currently remains the most promising approach to reduce the risk of T2DM in South Asian populations [15,16]. Indeed, available evidence indicates that interventions targeting diet and physical activity have the potential to prevent or delay the onset of T2DM for South Asian populations [17-19]. Further, we know that for these interventions to be maximally effective, they need to be culturally adapted to the specific needs and characteristics of the population or community they are aimed at, taking into account the diversity of populations and communities of South Asian origin, including factors such as ethnicity, gender, religion, language, and generation of migration [20,21].

Evidence indicates that text message interventions are effective in improving lifestyle risk factors, including diet and physical activity, and in reducing the risk of developing a range of chronic health conditions in the mainstream population [22,23]. Several recent studies found that text message interventions reduced the risk of T2DM in the mainstream population, and further studies demonstrated that text message interventions targeting factors such as diet and physical activity (along with medication adherence and education about the condition) were effective in secondary prevention in those who already developed T2DM in mainstream populations [24-26]. Text message approaches allow the development and implementation of interventions designed to meet the particular needs of a population or a community in a relatively straightforward, cost-effective manner compared with other approaches [22,23], and as such, text message approaches offer an advantageous means of providing culturally adapted interventions for specific South Asian populations. If proven successful, text message interventions can be easily scaled up and can be continuously available to individuals with minimum required investment—for example, through automated message delivery systems. Indeed, 2 recent studies in India provided evidence that lifestyle-focused text message interventions were effective in reducing the risk of T2DM in adult men with prediabetes [27] and in improving diet in the context of T2DM prevention in adults [28]. To the best of our knowledge, there is currently no text message–based T2DM prevention intervention specifically designed for South Asian populations living outside the Indian subcontinent.

Text message interventions targeting diet and physical activity in the context of preventing T2DM typically comprise a set of messages delivered to the recipient’s mobile phone over a period that aim to influence the recipient’s diet and physical activity, thus reducing their risk of T2DM. To provide culturally adapted text message–based interventions to prevent T2DM for South Asian populations, one needs to consider what the most relevant determinants of these health-related behaviors may be for the specific South Asian end user population and design messages to address these determinants, for example, incorporating specific cultural and social factors.

### Research Aims

This formative research aimed to develop a set of culturally adapted text messages to prevent T2DM for one specific South Asian population, women of Pakistani origin living in Scotland. The rationale for focusing on this subpopulation is that people of Pakistani origin are the largest ethnic minority group in Scotland where this study took place, with approximately 50,000 people identifying as Pakistani in the 2011 census [29]. Further, we recognize that the factors determining diet and physical activity and T2DM prevention are likely to differ between women and men from the same ethnic background [30-32] and, as there are persistent inequalities seen in the health of Pakistani women in the United Kingdom [33], we sought to address this historical lack of focus on their health needs.

## Methods

### Research Framework

**Figure 1:**
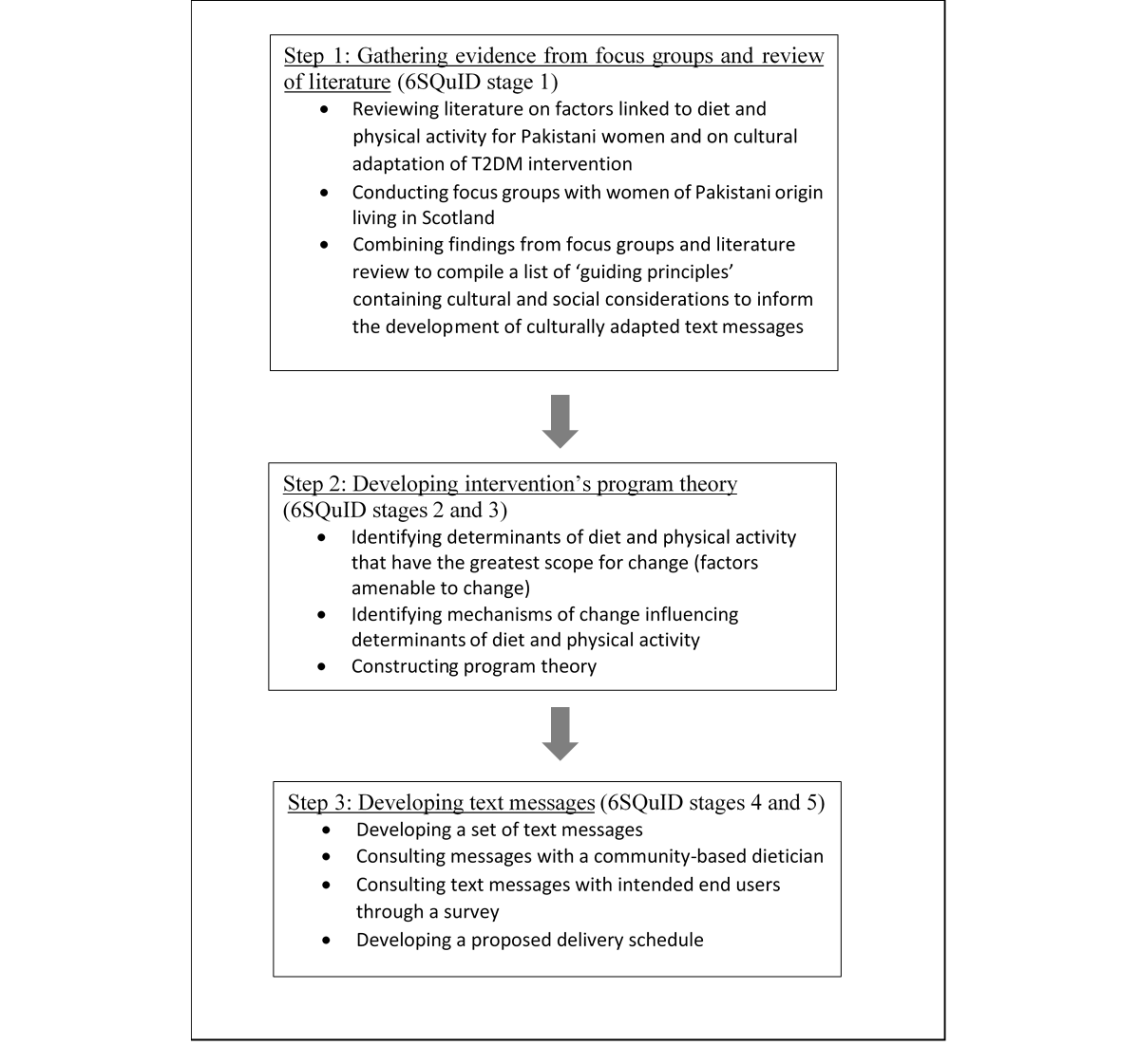
Outline of formative research to develop culturally adapted text messages to prevent type 2 diabetes mellitus (T2DM) for women of Pakistani origin living in Scotland. 6SQuID: Six Steps in Quality Intervention Development.

This formative research was conducted in 3 steps as outlined in Figure 1. Our approach was informed by the “Six Steps in Quality Intervention Development” framework [34]. This framework emphasizes an evidence-based, theory-informed, and pragmatic approach to intervention development to ensure the best possible effectiveness of the resulting intervention and it has previously been successfully applied to develop interventions for weight loss and lifestyle modifications [35,36]. We broadly followed stages 1 to 5 of the Six Steps in Quality Intervention Development framework, which we combined into 3 main steps (Figure 1).

This study formed part of “EuroDHYAN: Innovative Prevention Strategies for T2DM in South Asians Living in Europe” research project (grant 664609 HP-PJ-2014, European Commission Health Programme 2014-2020) of which the key aims were to advance the prevention of T2DM and to develop novel strategies to reduce the risk of T2DM for South Asian populations in Europe [37]. This project was grounded in the principles of culturally adapting interventions for ethnic minority populations and used, and built upon, an existing model of adaptation [20,21]. Other findings from the EuroDHYAN program have been reported elsewhere [15,16,20,38-40]. This study was conducted between January and October 2018.

### Ethics Approval

The ethical approval for this study was obtained from the ACCORD Medical Research Ethics Committee (REC reference: 17-HV-036), United Kingdom.

### Step 1: Gathering Evidence

#### Focus Groups and Narrative Review of Literature

Step 1 involved gathering and analyzing evidence on relevant factors affecting diet and physical activity in the context of T2DM prevention for women of Pakistani origin living in Scotland (ie, the intervention’s intended end users) and on assembling recommendations and insights on cultural adaptation of the text messages. In this step, we concurrently conducted a narrative review of the literature and a series of focus groups with women of Pakistani origin living in Scotland, and we describe the methods of these 2 pieces of work in the following subheadings.

#### Narrative Review of Literature on Factors Impacting on Diet and Physical Activity in the Context of T2DM Prevention

##### Aim of Narrative Review

The initial aim of this literature review was to collect evidence on the determinants (eg, cultural norms and beliefs and social practices) of diet and physical activity for women of Pakistani origin living in Scotland in the context of T2DM prevention. In the process of conducting the review, we discovered that the reviewed papers also discussed and stressed the importance of issues related to the cultural adaptation of T2DM prevention interventions to the needs of South Asian migrant populations. This prompted us to widen the original narrow focus of the review by adding an additional aim: to gather insights and recommendations on the cultural adaptation of T2DM prevention interventions for women of Pakistani origin living in Scotland (eg, eating practices related to specific types of food commonly consumed within South Asian communities, the role of acculturation as a result of living outside Pakistan, and gender and religious norms).

##### Eligibility Criteria

We included any publication from peer-reviewed journals published from inception to 2018 in English that analyzed the determinants of diet and physical activity in the context of T2DM prevention for women of Pakistani origin living in Scotland. During the process of screening for potentially eligible publications, we discovered that there were no studies that examined the determinants of diet and physical activity specifically for women of Pakistani origin living in Scotland. Therefore, to ensure that we collected rich and ample evidence, we decided to include any publication that reported on the determinants of diet and physical activity in the context of T2DM prevention for women of Pakistani origin living in Europe.

##### Search Process

We searched the Web of Knowledge and PubMed databases using the following search criteria: (“South Asian” OR “Indian American” OR “Pakistani” OR “Indian” OR “Sri Lankan” OR “Bangladeshi”) AND (“Diabetes”) AND (Prevent*“OR ‘Beliefs’ OR Norm*” OR “Cultur*”) AND (“Lifestyle” OR ‘Sport’ OR “Exercise” OR “Diet” OR “Food”), from inception to 2018. The studies were screened by 2 researchers (MK and EMD) to select a final set of papers according to the eligibility criteria, which would help to inform the development of the intervention.

##### Data Collection Process and Synthesis of Results

For each publication included in the review, we extracted insights on factors influencing diet and physical activity in the context of T2DM prevention and on cultural and social considerations when developing a culturally adapted T2DM prevention intervention for our intended end users. We then analyzed and synthesized the extracted evidence and focused on those determinants that can be addressed through text messages (eg, knowledge of healthy dishes) and omitted those determinants that were not likely to be effectively addressed through text messages (eg, wider social considerations and environmental factors such as lack of women-only gym facilities).

#### Focus Groups With Women of Pakistani Origin Living in Scotland

##### Aim of Focus Groups

The aim of the focus groups was to explore participants’ views on factors affecting their diet and physical activity in the context of T2DM prevention, views on factors that might impact participants’ increased risk of T2DM, ways to reduce the risk of T2DM through an intervention targeting diet and physical activity, and views on the use of mobile phones and text messages in preventing T2DM. We aimed to conduct at least 2 focus groups with 8 to 12 participants each and continued to conduct the focus groups until data saturation was achieved.

##### Selection of Participants

Adults women (aged ≥18 years) who self-identified as members of the Pakistani community in Scotland were included in the study. We aimed to include participants with or without a diagnosis of T2DM. Acknowledging the linguistic diversity of the Pakistani communities, we did not impose any language restrictions on our intended sample and included participants whose primary language was English, Urdu, Punjabi, or a combination of any of these languages.

We approached potential participants through a health care community link worker who put us in touch with a group of Pakistani women meeting regularly at a local community center. These women then agreed to host the focus groups during their weekly meeting and to advertise our project to the wider community in the area.

##### Data Collection

Each participant was given an information sheet (in Urdu or English, depending on their preference) and gave informed, written consent before the start of the focus groups. The focus groups were conducted with the help of a topic guide (Multimedia Appendix 1) by one of the researchers and by the health care link worker who also translated the researchers’ questions from English to Urdu and Punjabi when appropriate.

Each participant was offered a £10 (US $13) voucher as a token of appreciation for their time, and the researchers also covered the cost of a group meal served after the focus groups.

##### Data Processing and Data Analysis

All the focus groups were digitally audio recorded and transcribed verbatim, and the transcripts were anonymized. Sections of the transcripts that were in Urdu or Punjabi were then translated into English. We used an external agency specializing in providing transcription and translations for health care services and research to transcribe and translate the recordings. The transcripts were then analyzed using thematic analysis [41] and NVivo software (version 11; QSR International).

Finally, synthesizing the findings from the narrative review and the focus groups, we compiled a list of “guiding principles” to inform the development of culturally adapted text messages to address the specific needs of women of Pakistani origin living in Scotland.

### Step 2: Developing the Intervention’s Program Theory

#### Key Components of the Program Theory

The purpose of step 2 was to develop the text message intervention’s program theory that would outline, in a diagrammatic format, how the text messages aim to achieve the (intended) outcomes of improving diet and levels of physical activity for users of the intervention. We focused on developing two key components of the program theory: (1) a list of determinants of diet and physical activity (in the context of T2DM prevention for women of Pakistani origin living in Scotland) that had the greatest probability to be successfully targeted by a text message intervention and thus successfully bring about change for the intended end users—these determinants then become “short term outcomes” in the program theory; and (2) a list of change mechanisms, that is, mechanisms or techniques that would successfully target determinants of diet and physical activity and thus result in improved diet and levels of physical activity for the users of the intervention.

#### Formative Work Involved in the Development of the Intervention’s Program Theory

To select the determinants of diet and physical activity, we used insights from step 1. For example, we included cultural norms regarding meal preparation and knowledge on what constitutes a healthy diet as important determinants influencing diet and T2DM prevention for intended end users as these were factors identified through focus groups and the literature review. When developing the program theory, we aimed to build on the existing knowledge of how interventions targeting health behaviors (such as diet and physical activity) can support individuals in behavioral change. Instead of using one particular theory of behavior change, we based our approach on the behavior change techniques by Michie et al [42], which were developed to address the problem of there being numerous theoretical frameworks of behavior change and aimed to distill from these explicitly how to change the determinants of behavior. We mapped the factors influencing diet and physical activity, identified through the literature review, onto the list of 11 “types of determinants” of health behaviors (eg, knowledge and social support) from Michie et al [42]. We then selected those determinants for which there was supportive evidence for an impact on diet and physical activity in the literature review to be included in the program theory. For each determinant, we selected the most effective behavior change technique (or techniques) as recommended by Michie et al [42]. The selected behavior change techniques were then featured as mechanisms of change in our program theory. For example, we selected the behavior change technique (mechanism of change) “Prompts to seek social support” to address the determinant (factor amenable to change) “Peer support” featured in the program theory.

### Step 3: Developing Culturally Adapted Text Messages

#### Iterative Process of Developing Text Messages

In step 3, we aimed to develop a set of culturally adapted text messages focusing on diet and physical activity. Our intention was for these culturally adapted text messages to be the vehicles of the interventions’ mechanisms of change as outlined in the program theory. Culturally adapted text messages were developed through an iterative process that included drafting and redrafting of text messages by 2 researchers (EMD and MK) [28,43]. During the process of developing the text messages, we sought an existing text message intervention for diet and physical activity that was already embedded in service delivery for mainstream populations and had the potential to be adapted for ethnic minority populations. We found a web-based text message intervention (called “HealthyYouTxt” [44]) designed by the National Cancer Institute in the United States, which was freely available for people to sign up and aimed to help users toward a healthier lifestyle. These text messages had been originally developed for nonminority populations in the United States but had subsequently been culturally adapted for American Hispanic populations [43]. We contacted the team involved in developing this intervention who shared with us their “HealthyYouTxt” text messages for diet, physical activity, and weight loss, along with permission to use these to inform our intervention. We decided to use these text messages to supplement the development of our own text messages where appropriate.

In the process of developing culturally adapted text messages, we used the program theory developed in step 2 to select text messages that mapped on to the mechanisms of change, featured in the program theory, so that each text message represented ≥1 of the identified mechanisms of change (behavior change techniques). The process of adaptation was guided by the “Toolkit of adaptation” from Davidson et al [21], and to ensure that the text messages were culturally adapted for women of Pakistani origin living in Scotland, we followed the aforementioned “guiding principles,” generated from our focus groups and narrative review to prioritize which adaptations were most relevant and important for our proposed end users. We also used a range of publicly available materials on South Asian recipes to add further information to the text messages [38,45-49] and consulted with a South Asian community–based dietician on the content of the messages and implemented the minor changes she suggested into the revised version of the messages. We broadly intended for the set of text messages to be delivered over the course of 8 weeks, with 1 to 3 text messages sent out per day (this frequency had been selected based on the insights that emerged from step 1).

#### Feedback on Acceptability of Text Messages

Finally, we conducted a small-scale survey to collect suggestions for improvement and to assess the initial acceptability of the set of text messages. We approached women who took part in the focus groups to ask if they or any of their friends or relatives were interested in completing the survey. As part of the survey, each participant received a sample of about one-third of the total set of text messages (with different samples covering the whole set of messages) and was invited to provide any comments they may have on each individual text message (eg, whether they liked it, disliked it, or had any other comments). The survey also included 14 closed-ended and 4 open-ended questions regarding the entire set of text messages provided. The closed-ended questions focused on measuring aspects of the initial acceptability of messages, including the extent to which participants thought messages were “easy to understand,” “believable,” “relevant,” “made them think about their diet/physical activity,” etc. The answers were measured on a 4-point Likert scale (ie, “Strongly agree,” “Agree,” “Disagree,” and “Unsure”). We also asked participants to point out any messages that were difficult to understand, should be removed, or messages that they felt were inappropriate (and explain why if they wished). Finally, we asked for suggestions on how the messages could be improved. Each potential participant was mailed a stamped return envelope. Once we received a completed survey from a person, we mailed them a £10 (US $13) high street voucher to thank them for their time.

## Results

### Step 1: Gathering Evidence

#### Review of Relevant Literature

In total, we identified 12 peer-reviewed articles that met the inclusion criteria (Table 1 provides details of the reviewed papers) [32,40,50-59].

**Table 1:** Characteristics of 12 peer review articles included in the literature review.

Characteristics		Study, year
**Focus of the study (eg, diet and physical activity)**		
	Diet (in the context of T2DM^a^ prevention)	Lawton et al [56], 2008; Kjollesdal et al [58], 2010
	Physical activity (in the context of T2DM prevention)	Lawton et al [55], 2006; Sriskantharajah and Kai [50], 2007; Jepson et al [59], 2012
	Diet and physical activity (in the context of T2DM prevention)	Kjollesdal et al [57], 2011; Lucas et al [54], 2013; Morrison et al [52], 2014; Penn et al [51], 2014; Patel et al [32], 2017; Terragni et al [40], 2018
	Diet, physical activity, and obesity (in the context of T2DM prevention)	Ludwig et al [53], 2011
**Type of study**		
	Primary study (qualitative methods)	Lawton et al [55], 2006; Sriskantharajah and Kai [50], 2007; Lawton et al [56], 2008; Ludwig et al [53], 2011; Jepson et al [59], 2012; Morrison et al [52], 2014; Penn et al [51], 2014; Terragni et al [40], 2018
	Primary study (mixed methods)	Kjollesdal et al [57], 2011
	Primary study (quantitative methods)	Kjollesdal et al [58], 2010
	Systematic literature review	Lucas et al [54], 2013; Patel et al [32], 2017
**Population investigated**		
	Women of Pakistani origin living in England	Ludwig et al [53], 2011; Penn et al [51], 2014
	Women of Pakistani origin living in Norway	Kjollesdal et al [58], 2010; Kjollesdal et al [57], 2011
	Individuals of South Asian origin (including women of Pakistani origin) living in Scotland	Lawton et al [55], 2006; Lawton et al [56], 2008; Jepson et al [59], 2012; Morrison et al [52], 2014
	Women of South Asian origin living in England	Sriskantharajah and Kai [50], 2007
	Individuals of South Asian origin living in the United Kingdom	Lucas et al [54], 2013
	Ethnic minority populations in the United Kingdom (reporting distinct findings for women of South Asian origin)	Patel et al [32], 2017
	Researchers involved in developing and implementing T2DM prevention intervention for migrant South Asian populations	Terragni et al [40], 2018

^a^T2DM: type 2 diabetes mellitus.

We organized findings on factors affecting physical activity and diet in the context of T2DM prevention into 3 categories: knowledge, cultural and social norms, and sense of control or self-efficacy. These 3 categories broadly correspond with commonly identified determinants of health behavior change as described in modern theories of health behavior change and comprehensive classifications of determinants of health behavior [42].

Knowledge of ways to prevent T2DM through diet and physical activity was recognized as an important determinant in the reviewed literature. Studies reported several areas where lack of, or insufficient, knowledge was linked to poor diet and low levels of physical activity. This included lack of awareness that physical activity and healthy diet can help to prevent T2DM and lack of understanding of the link between lifestyle factors (such as diet and physical activity) and T2DM [50,54,57], not knowing how much and what type of physical activity is needed to help prevent T2DM [32,50,54], not having sufficient knowledge and information on what constitutes a healthy diet in the context of T2DM prevention (eg, how much salt or oil to use), and not having good healthy recipes to prepare dishes [32,58].

Cultural, social, and gender norms also featured as important determinants of diet and physical activity in women of Pakistani origin living in Europe and in South Asian migrant populations more generally in the reviewed literature. Specifically, norms related to social and cultural expectations to participate in frequent family and community gatherings where it is expected to prepare and share large quantities of food, including curries high in fat content, sweets, and soft drinks [32,51,52,56,58], and an expectation to prioritize the preferences of children, husbands, and other family members when preparing and consuming food (including an expectation to use large amounts of cooking oil) [32,52-54,56] were reported as barriers to healthy eating. Cultural expectations to prioritize responsibilities to kin over oneself and thus taking time to take part in organized exercise potentially being seen as selfish [32,54,55] and not being socialized to spend time outdoors on sport and physical activities [52,55] were reported as one of the key barriers to achieve higher levels of physical activity.

Finally, the reviewed literature also reported on the role of a lack of sense of control over one’s level of physical activity and diet and fatalistic beliefs about the causation of T2DM and a strong sense of inevitability [54,55].

#### Focus Groups

##### Collected Data

We conducted 3 focus groups, all of which took place in one community center in a single town in Scotland between January and April 2018. In total, 25 participants took part (n=8, 32% in focus group 1; n=8, 32% in focus group 2, and n=9, 36% in focus group 3). One focus group was conducted in English; 1 was conducted in Urdu (with some participants also speaking Punjabi); and 1 was conducted in a mix of Urdu, Punjabi, and English. The focus groups lasted from 60 to 90 minutes. Findings from these focus groups included what participants considered important relating to both the general attributes and also the more specific elements of the culturally adapted text messages.

##### General Attributes of Culturally Adapted Text Messages

Three attributes of culturally adapted text messages aimed at diet and physical activity and prevention of T2DM were identified as key to the success by focus groups participants.

We refer to these attributes as:

1. Friendly encouragement
2. Companionship
3. Focus on the individual

First, many participants spoke about “friendly encouragement” as central to any efforts aimed at changing their dietary habits or increasing the amount of exercise in their everyday lives. This was often discussed in the context of providing positive reinforcement and speaking with kind words while, at the same time, avoiding being judgmental or critical:

Encourage [women receiving text messages] but don’t make them feel bad about it.

Focus group 1

I think positive reinforcement, to be positive...if [text messages] are being negative, or you know, come on, get off your butt, that’s not the attitude to have.

Focus group 1

Second, participants often spoke about their wish for the text messages to provide a sense of companionship, which they felt would help with introducing changes to their lifestyle:

We want to receive a [text] message that lets us know that we are not alone in our lives.

Focus group 2

Even if it’s in your own home, you get the [text] message and might make you feel better and do something about it.

Focus group 1

Third, participants highlighted their wish for the text messages to focus on them as an individual and on their individual needs:

If I read a message you’re sending to everybody and even saying, for example, eat one apple every day, so then I will try, because somebody is focusing for me, for my lifestyle.

Focus group 1

This was often discussed using phrases, including: “me first” or “me time”:

Just a message saying, today’s me first. You are doing something for yourself for a change.

Focus group 1

Gender and social norms, including an expectation to prioritize the needs of others, such as family members over one’s own needs, were frequently discussed when participants raised their preference for the text messages to identify, legitimize, and prioritize their needs as an individual:

You see mother figure is the centre of the family, so she makes all the sacrifices for her whole family, and that’s why she just never thinks for herself...a lot of Asian women especially, they feel if they do something for themselves they’re being selfish, which I think is completely opposite.

Focus group 1

This expectation to prioritize the needs of others (over one’s own needs) was presented in opposition to being able to prioritize actions to improve one’s own health and well-being:

Because nine out of 10 times, we just do things for other people. Well, I am, it’s always my grandchildren or my son or my daughter needs this, never think of myself, so sometimes you have to sit and say, no, it’s me first.

Focus group 1

##### Specific Elements of Culturally Adapted Text Messages

Participants discussed the inclusion of two specific elements in the text messages:

1. Setting (achievable) goals
2. Providing information on cooking healthy meals

Many participants viewed setting achievable goals for changing diet and physical activity as a desirable element to include in text messages. These goals were intended to be introduced incrementally, so that they were perceived as achievable:

It’s about setting a goal for us, because you are to do the steps on it...so by starting not everything at once because if you start everything at once, then after a wee while you drop, because you’re fed up and you can’t do...you don’t have time.

Focus group 1

Second, participants pointed out that the text messages should provide information on what constitutes healthy cooking and healthy meals. In particular, the recommendation was to include information topics such as the use of cooking oils, salt content, and portion sizes, and recipes for healthy versions of popular South Asian dishes:

You could write something about healthy food, so people can cook this recipe.

Focus group 2

Participants also discussed issues related to the delivery of the messages, including length of the messages (“Small is better as we will be able to read it quicker and we will be able to act upon it.”*—*focus group 2) and frequency of delivery (“Once or twice per day. Not too much...because we do the house work etc., someone may visit when someone is trying to work. There is also the call to prayer and lots of other work to do.”*—*focus group 3).

#### Guiding Principles for Culturally Adapting the Text Messages

We synthesized insights from the narrative review and the focus groups to determine which dietary and physical activity behaviors were most important to target for women of Pakistani origin living in Scotland (Table 2) and to determine the most important cultural and social considerations (eg, culturally specific food and dishes and religious practices) when culturally adapting the intervention. These considerations were used as “guiding principles” in the process of culturally adapting the text messages and are outlined in Table 3, which also outlines how they align with guidance in the “Toolkit of adaptation.”

**Table 2:** Diet and physical activity targets of the intervention.

Behavior target	Specific subtargets
Promoting awareness that T2DM^a^ can be prevented	Addressing fatalistic beliefs about the causation of T2DM Addressing any sense of inevitability about getting T2DM
Promoting awareness of the link between diet and T2DM	Addressing any sense of lack of control over one’s diet Awareness of the many benefits of improving diet, including that it can help prevent T2DM
Promoting awareness of the link between a lack of PA^b^ and T2DM	Addressing any sense of lack of control over one’s levels of PA Awareness of the many benefits of increasing PA, including that it can help prevent T2DM
Providing information about how to improve diet^c^	Healthier food suggestions or substitutions Healthier food preparation techniques Awareness of snacking and promoting healthier snacks Awareness of sugar in drinks and promoting healthier drinks, for example, water Awareness of or reducing amount of carbohydrates in diet Awareness of or reducing amount of sugar in diet Awareness of or reducing amount of fat and oils in diet (including use of Ghee and deep-fried foods) Awareness of or reducing amount of salt in food (including how to flavor foods without using lots of salt) Portion size and a balanced diet Importance of fiber and increasing fiber in diet Addressing expectations to participate in gatherings where it is expected to prepare and share large quantities of food Addressing family expectations and preferences in food preparation
Providing information about how to increase PA and types of PA that can help prevent T2DM	Ideas for reducing sedentary behaviors Promoting aerobic exercise, for example, walking, cycling, and swimming Ideas for strength training Ideas for stretching How to build activities into daily life Suggesting exercise that can be done in the house (if weather and other obligations prevent going out to exercise, eg, family commitments, or if not being used to spending time outdoors) Promoting exercising with others, for example, walking group and fitness class Addressing cultural expectations to prioritize responsibilities to kin over oneself which may be a barrier to prioritizing exercise

^a^T2DM: type 2 diabetes mellitus.

^b^PA: physical activity.

^c^Where the focus groups and narrative review determined that more knowledge was needed on PA and diet, the extensive knowledge generated in the wider EuroDHYAN study [15,16,38-40] was used as evidence to determine the more specific subtargets.

**Table 3:** Guiding principles on cultural and social considerations for adapting the intervention.

Cultural and social considerations^a^	Typology of adaptation (adaptation number) or RESET tool question
Use encouraging, kind tone in text messages, avoid judgment and criticism (Diet and PA^b^)	Materials: material developed specifically for target population (23) Messages: guidance or messages base on preferences on preferences of target population (26) Delivery: addresses emotional barriers (42)
Focus on the need of the individual women receiving the messages prioritizing oneself within a context of responsibilities toward others, in the spirit of “me first” (Diet and PA)	Messages: guidance or messages based on preferences of target population (26) intervention content targets population’s social and cultural values (27) intervention considers issues unique to target population’s context (28)
Emphasize companionship aspects of text messages (Diet and PA)	Messages: guidance or messages based on preferences of target population (26) Delivery: addresses emotional barriers (42) encourages or involves social support (44)
Respect culturally relevant values, such as importance of family values (Diet and PA)	Messages: intervention content targets population’s social and cultural values (27)
Appreciate differences between women, do not treat the group as homogeneous. Do not overemphasize overall differences between ethnic and cultural groups at the expense of considering what other characteristics may be important to consider for people to feel the intervention resonates with their needs and circumstances (Diet and PA)	Messages: guidance or messages based on preferences of target population (26) RESET tool: Question 4: What elements of ethnicity are most important to consider for this population? (Heterogeneity)
Accommodate for more international food preferences (Diet)	Messages: guidance or messages based on preferences of target population (26) maintains cultural significance of food (34)
Promote changes to diet that work with current food practices and preferences, for example, by providing advice and recipes for dishes that use less fat. Start by introducing incremental changes to dietary choices (Diet)	Messages: guidance or messages based on preferences of target population (26) maintains cultural significance of food (34)
Focus on culturally relevant food, including sources of carbohydrates (eg, rice, roti, naan, and potatoes), vegetables (eg, courgettes, aubergines, and okra), pulses (eg, lentils and chickpeas), fruits (eg, mango and peaches), spices (eg, cinnamon, coriander, and salt), cooking oils (ghee, butter, and sunflower oil), main dishes (eg, biryani, nihari, pilau, and desserts [eg, kheer]), and drinks (eg, milk, tea, coffee, and fizzy drinks; Diet)	Messages: guidance or messages based on preferences of target population (26) maintains cultural significance of food (34)
Account for culturally relevant celebrations and gatherings, including the 4-week period of Ramadan (Diet)	Messages: intervention content targets population’s social and cultural values (27) intervention considers issues unique to target population’s context (28) maintains cultural significance of food (34)
Take into account external constraints, such as the cold Scottish climate, by including advice about the activities that can be performed indoors (PA)	Delivery: addresses physical and financial (structural) barriers to participation (43)
Encourage activities that are flexible and can be easily incorporated into women’s lives, capitalize on types of activities women already do in their everyday lives (PA)	Messages: intervention considers issues unique to target population’s context (28) Delivery: gender taken into consideration (46)
Advice for forms of physical activity that allows for social interactions (PA)	Delivery: encourages or involves social support (44)
Consider preferences for types of activities, such as walking and swimming and aqua aerobics (with “women only” facilities/hour), exercises that can be done indoors or at home (eg, walking up and down the stairs, house work; PA)	Messages: guidance or messages based on preferences of target population (26) Delivery: gender taken into consideration (46)

^a^The cultural and social considerations comprised guidance on developing lifestyle advice (comprising Diet and PA) in general and guidance on developing text messages focus on diet or PA in particular.

^b^PA: physical activity.

### Step 2: Developing Program Theory

**Figure 2:**
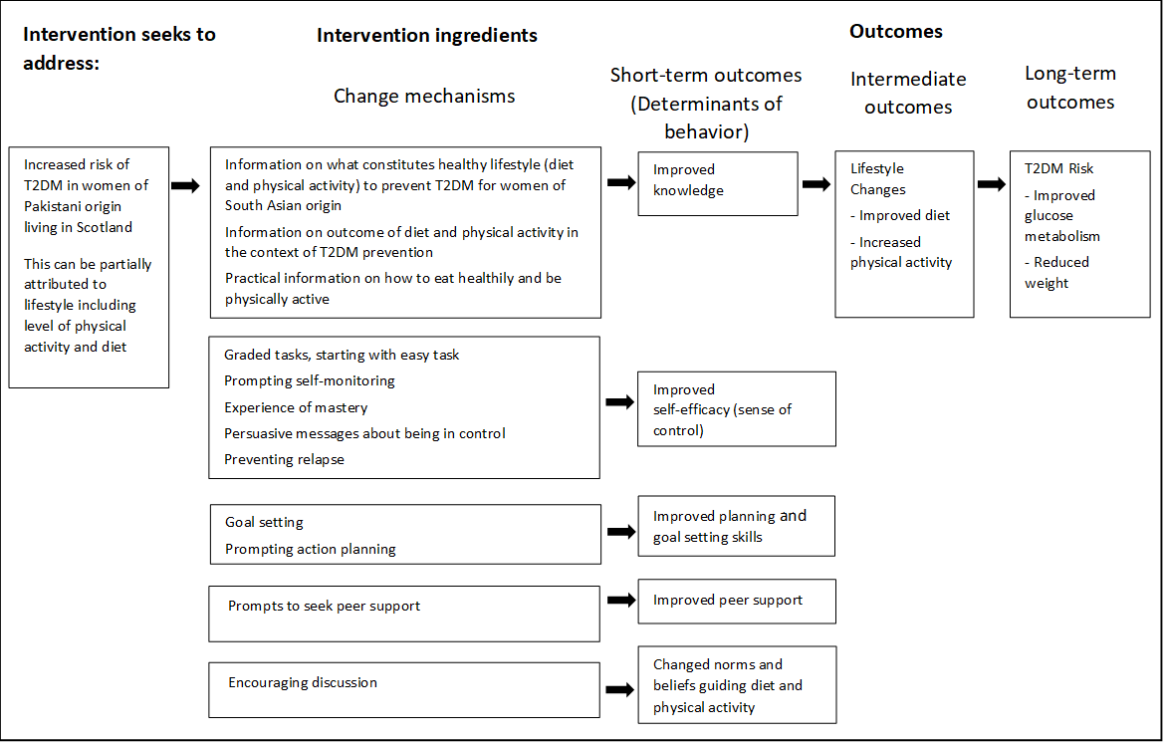
Program theory for the intervention. T2DM: type 2 diabetes mellitus.

Figure 2 outlines the intervention program theory and is composed of the following elements: (1) the description of the problem the intervention seeks to address; (2) a list of mechanisms of change through which the intervention aims to achieve its intended outcomes; and (3) a list of outcomes, including short-, intermediate-, and long-term outcomes. The determinants of behavior included in the program theory are presented under the heading “short-term outcomes.” We ultimately selected 5 categories of determinants of diet and physical activity that the text messages sought to address: “Knowledge,” “Self-efficacy” (or “Sense of Control”), “Action Planning” (or “Goal Setting”), “Social Norms and Beliefs,” and “Social Support” (Table 4 provides a detailed description of how insights from step 1 informed the selection of these 5 determinants).

**Table 4:** Insights from step 1 of this formative research (focus groups and narrative literature review) that informed selection of 5 determinants of diet and physical activity in the context of type 2 diabetes mellitus (T2DM) prevention featured in the intervention’s program theory.

Determinants	Step 1 insights
Knowledge of ways to help prevent T2DM through diet and physical activity	Focus groups: Providing information on cooking healthy meals featured as 1 of the 2 specific elements of culturally adapted text messages requested by focus group participants. Literature review: Literature review highlighted lack or insufficient knowledge on what constitutes a healthy diet and the right amount and type of physical activity in the context of T2DM preventions as well as insufficient knowledge of link between lifestyle and risk of developing T2DM as associated with diet and physical activity.
Self-efficacy; sense of control over one’s diet and physical activity and sense of inevitability about developing T2DM	Focus groups: “Friendly encouragement” was one of the general characteristics of culturally adapted text messages requested by focus groups participants. The “friendly encouragement” can be interpreted in the context of enhancing person’s belief that they can get through the steps necessary to achieve the desired outcomes and have necessary skills and resources to deal with situations and challenges they face. This in turn corresponds with the concept of self-efficacy and also to an extend sense of control as described in psychology literature. Literature review: Lack of sense of control over one’s diet and level of physical activity was featured as one of the factors associated with diet and physical activity in relation to T2DM prevention. Sense of inevitability about developing T2DM was also featured as an important factor in the context of T2DM prevention.
Planning skills, including goal setting	Focus groups: “Setting achievable goals” featured as 1 of the 2 specific elements of culturally adapted text messages requested by focus group participants. In addition, “friendly encouragement” was featured as 1 of the 3 general attributes of culturally adapted text messages request by focus group participants. These correspond with the concept of self-efficacy or sense of control as it focuses on supporting the individual in developing believe that they can perform the desired actions and behaviors. Literature review: No direct evidence on the role of action planning or goal setting emerged from the literature review.
Norms and beliefs guiding diet and physical activity	Focus groups: Gender and social norms relate to an expectation to prioritize the needs of others such as family members were discussed by focus groups participants. This was in the context of focus groups participants asking for the text messages to their individual needs and priorities (eg, “me first” or “me time”). Literature review: Social and gender norms with regard to such aspects of day-to-day life as preparing meals at home, attending social and family gatherings, and organized exercise have been strongly featured as impacting on diet and physical activity in the context of T2DM prevention in the literature review.
Peer support and companionship	Focus groups: “Companionship” was 1 of the 3 general attributes of culturally adapted text messages request by focus group participants. This meant that the text messages were meant to serve as form of companionship to its users (having someone supporting them in their journey of behavior change), but also more generally supported people in seeking companionship and peer support in the context of diet and physical activity. Literature review: Having a companion for physical activity and exercise and the physical activity providing an opportunity for socializing was identified as an important factor in the context of increasing physical activity in the literature review.

### Step 3: Developing Culturally Adapted Text Messages

We developed a set of 138 text messages, of which 65 (47.1%) focused on physical activity and 73 (52.9%) focused on diet. Examples of text messages for diet and physical activity addressing each of the determinants of lifestyle featured in the program theory are presented in Table 5 (Multimedia Appendix 1 provides a full list of text messages). We broadly aimed for the text messages to be delivered over the course of 8 weeks. The text messages about diet and physical activity were designed so that they could be delivered in parallel over the same period or separately. The topics covered by text messages for diet included fiber, carbohydrates, sugar, fat, salt, drinks, snacks, portion sizes, and celebrations. For text messages focusing on physical activity, the topics covered included increasing physical activity, decreasing sedentary lifestyle, and building strength. For both the text messages focusing on diet and physical activity, we divided the messages into 2 broadly equal parts, with the first part focusing on introducing changes to diet and lifestyle activity and the second part focusing more on maintenance and relapse prevention. To address the specific request that emerged from the focus groups about “setting achievable goals,” we included a subset of text messages dedicated to “Weekly challenges.” We set 4 weekly challenges for diet and physical activity (Table 5). For each text message, a person receiving the text messages could decide whether they wanted to take the challenge by replying “YES” or “NO” to the text message. If the person replied “YES,” they would receive a set of text messages at the end of the week to check how they were doing. We aimed for the text messages dedicated to weekly challenges to address several of the mechanisms included in the program theory: the “goal setting” as well as “graded task, starting with easy task” (by starting with setting relatively easier challenges in the first week and then progressing to more difficult challenges) and persuasive messages about being in control.

**Table 5:** Examples of text messages constituting the intervention.

Mechanism of change (behavior change technique) conveyed by the text message	Text messages about “Diet”	Text messages about “Physical activity”
Information on what constitutes healthy lifestyle (diet and physical activity) to prevent T2DM^a^ for women of South Asian origin	*Diet* “You can use fresh herbs and spices instead of salt or high sodium sauces to flavour your food. Too much sodium can lead to high blood pressure, heart disease, stroke and kidney disease.”	*Physical activity* “Physical activity can include both daily activities—such as walking, climbing stairs or housework/cleaning, or organised activities like joining a walking group, taking part in a swimming or yoga class.”
Information on outcome of diet and physical activity in the context of T2DM prevention	*Diet* “Eating healthily has immediate benefits of giving you more energy for your day to day tasks and improving your sleeping pattern and it can also help to delay the onset or reduce the risk of developing type 2 diabetes and cardiovascular disease and will improve your health overall!”	*Physical activity* “Sitting too much has been shown to be bad for your health, even if you exercise at other times, and is linked to an increased risk of diabetes. You will benefit from reducing the time you spend sitting each day, and from breaking up periods of time spent sitting, as often as possible.”
Practical information on how to eat healthily and be physically active	*Diet* “More examples of healthy snacks you can enjoy alone or with others includes: samosas baked in the oven, plain puffed rice with spices, dry roasted chickpeas or oatcakes. You can pick one of these or create your own healthy snack that works for you and your family!”	*Physical activity* “Particularly at the weekend, parenting/family commitments can make it hard to take part in organised exercise? Rethink exercise! Be active with your family—head into the garden or to a park to get in your fitness time instead.”
Graded task, starting with easy task	*Diet* “If abandoning all fizzy and sugary drink all together feels like too much you can try to reduce the amount you drink gradually. Remember: it is the long-term change that counts!”	*Physical activity* “Get your heart pumping with some activity like brisk walking, biking or dancing. Start where you are and try to build up gradually to the 30 min/day.”
Prompting self-monitoring	*Diet* “You can try keeping a journal of all the snacks you have eaten during the day. It can help you with knowing exactly what you eat and with planning changes you want to make.”	*Physical activity* “Keep note of how much time you spend sitting each day, what you’re doing when sitting (for example watching TV, reading or working), and plan how you could make small changes to gradually reduce this time or bring some activity into this time.”
Experience of mastery	*Diet* “By receiving this message you have already helped to protect your long-term health and reduce the risk of type 2 diabetes in the future. Stay with us for second half (Week 5-8) to strengthen your skills and help prevent diabetes.”	*Physical activity* “Great job! Finding ways to sit less & move more is a big step toward an active, healthy lifestyle. Keep it going next week!”
Persuasive messages about being in control	*Diet* “You can have a lot of control over how much unhealthy fats (ghee, butter, full fat milk) and deep-fried food you eat and prepare!”	*Physical activity* “Hope you enjoyed the messages about increasing physical activity and that you feel more in control of what steps you can take to get more active.”
Preventing relapse	*Diet* “Hope you enjoyed this week’s text-messages! You should not get discouraged if you don’t get everything right all the time. Making changes takes time!”	*Physical activity* “A tip for the future: Small lapses are not going to undo all the changes you have made. Remember to stay positive about the changes you have made already.”
Goal setting	*Diet* Week 2 Challenge: “Reducing the amount of unhealthy snacks is an easy way to make healthy changes to your diet right away! Try reduce the amount of sweet or salty snacks you eat this week. Do you want to take part in this week’s challenge? Reply YES or NO.” *Diet* “Good luck! We will get back to you at the end of the week to check how you were doing.” (If replied “Yes” to the first text message) *Diet* “That is all right! We hope you will enjoy the messages we will send you think week.” (If replied “No” to the first text message) *Diet* Week 2 Challenge check in: “Were you able to reduce the amount of salty and sweet snacks this week? Reply YES or NO.” *Diet* “You are doing a great job! Cutting down on unhealthy snacks has a positive impact on your diet and helps minimise the risk of diabetes in the future. Try to think about strategies that worked for you and keep at it!” (If replied “Yes” to the second text message) *Diet* “Don’t worry! Every small change is a step in the right direction! Try to think about strategies that worked for you and keep at it!” (If replied “No” to the second text message)	*Physical activity* Week 3 Challenge 3: “Try to plan more exercise this week and each week following. For example, this week: Be active for 10 minutes on 3 days of the week. Next week: 10 minutes on 4 days. The following week: 15 minutes on 3 days, and so on. We are aiming for the goal of 30 minutes of activity a day. Do you want to take part in this week’s challenge? Reply YES or NO.” *Physical activity* “Fantastic! We will get back to you at the end of the week to see how you were doing.” (If replied “Yes” to the first text message) *Physical activity* “That’s alright. You can still read this week’s messages and see if you find them helpful.” (If Replied “No” to the first text-message) *Physical activity* Week 3 Challenge check-in: “You made it through Week 3! Were you able to get more active this week? Reply: YES or NO.” *Physical activity* “Great job! Finding ways to sit less & move more is a big step toward an active, healthy lifestyle. Keep it going next week!” (If replied “Yes” to the second text message) *Physical activity* “Some ups & downs are normal. Use next week’s challenge as motivation to keep moving forward toward an active lifestyle.” (If replied “No” to the second text message)
Prompting action planning	*Diet* “Changing habits is not easy, but even small changes are a step in the right direction. Think about what you might do next week to cut back on fats.”	*Physical activity* “Do you do a lot of driving? Next time you’re going somewhere close, try walking—it’s good for your body and the environment”
Prompts to seek social support	*Diet* “Tip for this week: Why not plan a healthy meal with your family and people close to you to celebrate the end of the programme! What would you make?”	*Physical activity* “It may help to think of a form of exercise that suits your interests and perhaps even a hobby you could take up that would keep you more active. Is there something you can do with a group of friends or family to support each other to keep active?”
Encouraging discussion	*Diet* “Sometimes it might be difficult to have a control over how much you eat. For example, it is common in South Asian culture that during family gatherings when it might be expected to take second helpings or give or receive sweets. There is no easy solution to that, but if that is the case for you, can you think of ways in which you can regain control over how much you eat? You can try talking to your friends and people close to you about it. Two heads are better than one!”	*Physical activity* “The weekend is a really good chance to get up and out, and to be active with family and friends. Try to find out what people close to you are doing to keep active, or would like to do and plan to be active together.”
Example of a text message that could have a link added to provide users with ideas and resources available locally	*Diet* “Are you thinking about the menu for the weekend? Why not try low-fat dishes, such as baked samosas, khasta roti made without oil or milk pudding made with low fat milk. You can also make your own low recipe and try it out this weekend!” (Could include link to low-fat recipes)	*Physical activity* “If you are getting bored with your current exercise activities, make plans to try something new. Is there a walking group you could join, a fitness or swimming class or a fitness dvd you could use at home?” (Could include link to appropriate local venues)

^a^T2DM: type 2 diabetes mellitus.

We consulted on the draft text messages with 7 women among those who participated in the focus groups. All 7 participants returned the questionnaire and 3 left written feedback in relation to the particular messages. All 7 women “Agreed” or “Strongly agreed” to the following statements about the sample messages that they received attached to the survey: “easy to understand,” “believable,” “felt as if they were from someone I could trust,” “relevant,” “effective,” “taught me something new,” “made me stop and think,” “made me feel concerned about my diet,” “made me feel concerned about my levels of physical activity,” “motivated me to change my diet,” “motivated me to change my level of physical activity,” “felt respectful,” and “made me want to talk to other people about the messages.” All 7 women disagreed with the statement that the messages “felt like they talked down to me.” None of the 3 women who also completed the second part of the survey indicated that any of the text messages attached to the survey were “difficult to understand,” “felt inappropriate,” or “should be removed.” None of the women participating in the survey suggested introducing any changes to the text messages. Two themes were presented in the free text response: a view that the messages were framed in a positive language which was appreciated (eg, “Great positive message.” and “Love the positive vibes from this text-messaging services. Will motivate people.”) and a view that the messages were written in a simple accessible language which again was appreciated (eg, “Explains very well, simple message.” and “Reducing [fizzy drinks] is a way forward. Very well explained.”). We also received positive comments about the text messages describing the “Weekly challenge” (eg, “Great challenge, keeps people interested.” and “Love the motivation.”).

## Discussion

### Principal Findings

In this formative, theory-informed [34] research, we developed a set of culturally adapted text messages aiming to support women of Pakistani origin living in Scotland in preventing T2DM through diet and physical activity. The text messages were designed to be delivered over an 8-week period with a possibility to deliver “Diet” and “Physical activity” components over the same period or separately. The focus groups, conducted at the early stages of this formative work, provided guidance regarding views of women of Pakistani origin living in Scotland on preferred characteristics of text message–based interventions to prevent T2DM. This included preference for text messages worded in a tone conveying friendly encouragement and avoiding being judgmental or critical, providing a sense of companionship, and focusing on the needs of the individual in the context of responsibilities toward others, as well as including setting achievable goals and information on cooking healthy meals as part of the intervention. The narrative review identified important factors influencing T2DM prevention on an individual level, including insufficient knowledge on ways to prevent T2DM through diet and physical activity; social, cultural, and gender norms; perceived lack of control over one’s diet and physical activity; and a sense of inevitability about developing T2DM. Finally, based on the narrative review and focus groups, we formulated guiding principles for culturally adapting text messages for our intended end users and created a program theory for the intervention. The above findings informed the development of a set of culturally appropriate text messages and offer the potential to help inform the development of future T2DM prevention interventions for minority populations.

### Interpretation in the Context of Wider Literature

To our knowledge, our formative work represents a first attempt to develop a text message–based T2DM prevention intervention for South Asian populations living outside the Indian subcontinent, which specifically takes into account the diversity of this population and the specific nuances of their context. A recent review, also carried out as part of the EuroDHYAN project [37], exploring the theoretical underpinning of culturally adapted interventions suggests that consideration of heterogeneous population characteristics and the broader contexts in which people are living can certainly be pivotal in determining whether intervention mechanisms of change trigger their intended outcomes [20].

Literature examining the use of text messaging interventions for T2DM prevention for South Asian populations is relatively scarce. At the time of developing our intervention, we found 2 existing studies, both conducted in India, which demonstrated that text message–based interventions could be an effective strategy. A study by Ramachandran et al [27] used a text message–based approach to target lifestyle factors for adult South Asian men with prediabetes (employees of major industries) living in India and reported that their intervention resulted in a 36% relative reduction in the incidence of T2DM after 2 years. In the second study, Pfammatter et al [28] randomly recruited participants from adult Nokia subscribers in India and reported that their text message–based intervention (mDiabetes Arogya) was effective in improving diet, but not physical activity 6 months later. The mDiabetes Argoya intervention participants comprised a very heterogeneous population situated across the rural and urban areas of India, with messages delivered in 12 languages.

As our study was completed, 2 further relevant studies have been published. A 3-arm cluster-randomized trial (DMagic) compared a mobile health (mHealth) intervention (using recorded voice messages rather than text messages), participatory community mobilization, and usual care for T2DM prevention in 96 villages in Bangladesh [60]. The study found that the mHealth intervention increased knowledge and awareness of T2DM but did not influence disease outcomes for their participants. Nanditha et al [61] reported on an extension of the successful intervention initially evaluated by Ramachandran et al [27] in India, which included both adult women and men with prediabetes and was conducted simultaneously in both India and the United Kingdom [61]. The design and content of the messages used in their previous study [27] were modified and expanded, including with input of a patient and public involvement group in the United Kingdom. However, evaluation at 2 years showed that the effectiveness of their previous study was not replicated and there was no significant difference in the progression to diabetes between the intervention and control group, a result that has been partially attributed to the significant heterogeneity of the intervention’s recipients and their sociocultural contexts. They recommended that the influences of these differing contextual factors on the effectiveness of this type of intervention warrants further investigation.

A recent qualitative study presented the first report of the exploration of the views of the British South Asian population on this type of text messaging intervention designed for the mainstream population to support T2DM medication adherence [62,63]. The results of these exploratory focus groups with a diverse sample of South Asian participants living in Leicester, United Kingdom (including Indian Punjabi Sikh, Pakistani Muslim, Indian Gujarati Hindu, Bangladeshi Muslim, and Indian Gujarati Muslim participants of both the first and second generation) showed that their study population was, in addition to medication adherence, also interested in receiving messages relating to lifestyle factors such as diet and physical activity. They also reported that, for this intervention to meet this population’s needs, it should include culturally adapted messages for those who would like to receive them. The findings from focus groups conducted as part of this formative research add to these findings by highlighting a strong call for messages to be encouraging and kind, avoiding judgment and criticism, and focusing on the individual in a way that helps people prioritize their needs and make time to take care of their health. Incorporating these findings into the tone of our messages enabled us to create an atmosphere that appeared to appeal to, and fit the needs of, our participant population.

There is also minimal literature on the process of culturally adapting text messages for physical activity and diet, although we have discovered work undertaken in 2 different settings, including for Hispanic adults in the United States [43] and, following the completion of our work, for Māori and Pasifika Communities in New Zealand [64]. Both of these works emphasized taking a systematic and theory-driven approach to adaptation, using behavior change theory and assessing whether techniques are appropriate for the population of interest, and also stressed the importance of involving the community closely in intervention development. A similar process has been used in New Zealand for the development and adaptation of text messaging interventions for other health programs, including maternal health promotion [65], and these are based on an underlying mHealth Development and Evaluation framework that also emphasizes these core principles [66].

### Strengths and Limitations

This study had several key strengths. First, we undertook detailed formative work, including consulting the intended end users through a series of focus groups and a short survey. Second, we used established frameworks of intervention development [34], behavior change techniques [42], and cultural adaptation [21] to guide this formative work and developed a program theory (detailing the factors amenable to change targeted by the interventions, the mechanisms of change addressing those factors, and the intended outcomes) rather than launching headlong into a poorly theorized trial. Such rigorous development work reflects the core principles of the wider work we observed in New Zealand [64] and the United States [43] and helps to ensure that the intervention meets the needs of the populations it is designed for, ensuring the best possible future effectiveness of the intervention. Third, careful reporting of the development process helps to understand the underlying theory and guide future work on developing similar interventions. Our intervention messages were developed for the context of our specific population and, as such, would not be directly generalizable for use by other populations without further formative work or assessment of their suitability by the intended end users. However, a key strength of our approach to the development of this intervention and our detailed reporting is that they enable this process to be openly transferrable to other health areas, populations, and population contexts.

One of the main limitations is that we were only able to identify a limited body of literature focusing on determinants of diet and physical activity for women of Pakistani origin living in Scotland. We partly addressed this limitation by including publications that reported on those factors for women of Pakistani origin living in other parts of the United Kingdom and in other European countries. Second, although we conducted 3 focus groups in total, most insights regarding the views on general attributes of culturally adapted text messages came from focus group 1, which was conducted entirely in English as opposed to the other 2 focus groups that were conducted either in a mix of English, Urdu, and Punjabi or in Urdu and Punjabi only. It is possible that the use of an interpreter in the other 2 focus groups disrupted the process of qualitative research. This highlights the challenges of conducting research in the context of multilingual populations [40]. Perhaps, having a multilingual qualitative researcher conducting the focus groups could have addressed this shortcoming. Third, we were only able to obtain a small number of participants for the small-scale survey to consult text messages and ask for suggestions for improvement from the intended end users, thus potentially limiting the input of the intended end users on the final wording of the text messages. Fourth, a potential limitation was that we developed text messages in English language only, but not in Urdu and Punjabi, which are spoken by a proportion of our intended end users. A recent study by Prinhja et al [62] with British South Asian people indicated that participants were happy to receive messages in English and that they felt they could have help from family members to translate these if needed. Fifth, another potential limitation of using text messages to deliver the intervention is how differing levels of health literacy may influence how these messages are processed, and this may be of particular concern for ethnic minority populations [67,68]. Finally, although this is not directly a limitation of this formative research, we acknowledge that only a section of the determinants of diet and physical activity and other risk factors for developing T2DM can be addressed through an intervention consisting of text messages sent to individuals at risk of developing T2DM. Wider societal considerations such as lack of appropriate gym facilities and health inequalities need to be addressed through other means.

### Implications for Research

The next stage for this work is to undertake a formal feasibility trial. If successful, this will then need to progress to a pilot and eventually to a definitive randomized controlled trial [69]. We propose that this would best progress embedded within the existing health care system. Rather than continuing this in a purely research setting, this would promote the sustainability of the intervention and try to mitigate the negative impacts on communities that have been recognized to arise when research-based interventions come to an end and no ongoing program is available [20]. For the same reason, we would recommend embedding this intervention within the wider program of support and resources available for T2DM prevention within a health care system as successful prevention of T2DM within populations requires ongoing rather than one-off efforts. For example, we propose that the 8-week text message–based intervention described in this paper could be followed by another, less intensive text message–based intervention where individuals would receive text messages focused on sustaining T2DM prevention over a longer period, but with a lower frequency of text messages received per week. Future efforts may also wish to consider exploring the concomitant or alternative use of newer technologies to enhance the intervention or the possibility of further tailoring the intervention to be responsive to individuals, for example, including a baseline assessment or data gathering segment at the start of the intervention, which could be used to determine which sequence of messages participants receive. Finally, we propose that future studies can investigate whether this text message intervention can be successfully translated into other contexts, including adapting the intervention for other South Asian populations.

Furthermore, in this paper, we presented a structured, stepwise, theory-informed approach for the development of a culturally adapted text message T2DM prevention intervention. Our approach could be helpful for researchers wishing to develop text messaging interventions for other populations or conditions and in diverse settings.

### Conclusions

In summary, this formative work resulted in developing a culturally adapted diet and physical activity text message intervention to prevent T2DM for women of Pakistani origin living in Scotland. In the next step, this text message T2DM prevention intervention should be tested in a feasibility trial and ultimately in a pilot and definitive randomized controlled trial to test its effectiveness. If such a trial is successful, this approach could provide a cost-effective way to deliver a T2DM prevention intervention at scale for women of Pakistani origin living in Scotland. It also presents a model that could be used to adapt other text message interventions for other populations and diverse contexts.

## Appendix

Multimedia Appendix 1 Topic guide and full set of text messages.

## Abbreviations

mHealth: mobile health
T2DM: type 2 diabetes mellitus

## Data Availability

Data are largely included in the manuscript or provided in Multimedia Appendix 1.
